# Cost-effectiveness analysis of three algorithms for diagnosing primary ciliary dyskinesia: a simulation study

**DOI:** 10.1186/s13023-019-1116-3

**Published:** 2019-06-13

**Authors:** Panayiotis Kouis, Stefania I. Papatheodorou, Nicos Middleton, George Giallouros, Kyriacos Kyriacou, Joshua T. Cohen, John S. Evans, Panayiotis K. Yiallouros

**Affiliations:** 1 Respiratory Physiology Laboratory, Medical School, University of Cyprus, Nicosia, Cyprus; 2 Cyprus International Institute for Environmental and Public Health, Cyprus University of Technology, Limassol, Cyprus; 3 Department of Epidemiology, Harvard T.H. Chan School of Public Health, Harvard University, Boston, MA USA; 4 Department of Nursing, Cyprus University of Technology, Limassol, Cyprus; 5 Department of Electron Microscopy and Molecular Pathology, Cyprus Institute of Neurology and Genetics, Nicosia, Cyprus; 6 Cyprus School of Molecular Medicine, Nicosia, Cyprus; 7 Tufts Center for the Study of Drug Development, Boston, USA; 8 Department of Environmental Health, Harvard T. H. Chan School of Public Health, Boston, USA; 9 Shiakolas Educational Center of Clinical Medicine, Palaios Dromos Lefkosias-Lemesou 215/6, 2029 Aglantzia Nicosia, Cyprus

**Keywords:** Primary ciliary dyskinesia, Diagnosis, Cost-effectiveness analysis, Decision analysis Kartagener syndrome, Nitric oxide, High speed video microscopy, Transmission Electron microscopy

## Abstract

**Background:**

Primary Ciliary Dyskinesia (PCD) diagnosis relies on a combination of tests which may include (a) nasal Nitric Oxide (nNO), (b) High Speed Video Microscopy (HSVM) and (c) Transmission Electron Microscopy (TEM). There is variability in the availability of these tests and lack of universal agreement whether diagnostic tests should be performed in sequence or in parallel. We assessed three combinations of tests for PCD diagnosis and estimated net sensitivity and specificity as well as cost-effectiveness (CE) and incremental cost-effectiveness (ICE) ratios.

**Methods and results:**

A hypothetical initial population of 1000 referrals (expected 320 PCD patients) was followed through a probabilistic decision analysis model which was created to assess the CE of three diagnostic algorithms (a) nNO + TEM in sequence, (b) nNO + HSVM in sequence and (c) nNO/HSVM in parallel followed, in cases with conflicting results, by confirmatory TEM (nNO/HSVM+TEM). Number of PCD patients identified, CE and ICE ratios were calculated using Monte Carlo simulations. Out of 320 expected PCD patients, 313 were identified by nNO/HSVM+TEM, 274 with nNO + HSVM and 198 with nNO + TEM. The nNO/HSVM+TEM had the highest mean annual cost (€209 K) followed by nNO + TEM (€150 K) and nNO + HSVM (€136 K). The nNO + HSVM algorithm dominated the nNO + TEM algorithm (less costly and more effective). The ICE ratio for nNO/HSVM+TEM was €2.1 K per additional PCD patient identified.

**Conclusions:**

The diagnostic algorithm (nNO/HSVM+TEM) with parallel testing outperforms algorithms with tests in sequence. These findings, can inform the dialogue on the development of evidence-based guidelines for PCD diagnostic testing. Future research in understudied aspects of the disease, such as PCD-related quality of life and PCD-associated costs, is needed to help the better implementation of these guidelines across various healthcare systems.

**Electronic supplementary material:**

The online version of this article (10.1186/s13023-019-1116-3) contains supplementary material, which is available to authorized users.

## Introduction

Primary Ciliary Dyskinesia (PCD) is a genetically heterogeneous disorder that affects one in approximately 15,000 live births [1]. PCD is characterized by chronic sinopulmonary symptoms and development of bronchiectasis, recurrent otitis, male infertility and situs inversus [2]. Defective components of the ciliary axoneme (e.g. dynein arms) as well as dysfunctional regulatory or transport proteins have been implicated in the etiology of PCD and to date more than 40 genes have been found to be causative for PCD [3]. This genetic heterogeneity translates into a wide spectrum of ciliary structural and beating abnormalities and a diverse diagnostic and clinical phenotype. Patients with PCD usually present with chronic cough and rhinorrhea as well as recurrent infections of unknown aetiology. Some of them also present with situs abnormalities and in the case of older patients, with infertility or subfertility [2]. Bronchiectasis may develop already in childhood in some patients [4] and it is usually present in most adult PCD patients [5]. Late diagnosis is associated with a worse clinical picture and reduced lung function [6, 7], while several patients undergo surgical resection of lung segments to control lung infection, even before diagnosis is established [8]. Situs inversus is the only characteristic manifestation associated with PCD. With the exception of chronic cough and rhinorrhea, all other manifestations may not always be present and may be characterized by considerable variability in their severity [9–11]. As a result, heterogeneity in the clinical picture presents a challenge to the clinician who needs to decide when to test for PCD and with which diagnostic test(s). Diagnostic approach is further perplexed by heterogeneity in the diagnostic features of the disease as respiratory epithelial samples from PCD patients exhibit diverse ciliary ultrastructure [12] and motility pattern [13] especially in the presence of infection [14].

Up to date diagnostic testing for PCD relies on a combination of tests which primarily includes nasal Nitric Oxide (nNO) [15], High Speed Video Microscopy (HSVM) [16, 17] and Transmission Electron Microscopy (TEM) [8, 18]. Measurement of nNO is considered the simplest and fastest among the PCD diagnostics tests as it only involves air suction from the nasal passage via an olive while the subject preferably maintains velum closure through active mouth exhalation against resistance [19]. The other two tests require brushing of the inferior nasal turbinate and the collection of an adequate sample of respiratory epithelial cells in order to allow for the assessment of ciliary motility using HSVM and ciliary ultrastructure using TEM [20]. As no single test has 100% sensitivity and specificity [21], which is further complicated by the fact that many centers lack either the equipment or expertise to perform all required tests, some of which are quite laborious and time consuming, different diagnostic algorithms for diagnosis of PCD have been adopted by diagnostic centers across the world [22]. Recently, nNO has been proposed as the screening test of choice in cohorts of patients with PCD-suspect manifestations due to its high ability to discriminate between PCD and non-PCD subjects [15, 23]. Although the cost of a (validated) chemiluminescence NO analyser is quite high (approximately €40,000 per piece), the recent development of handheld and cheaper electrochemical NO analysers [24] and publication of relevant technical guidelines by the American Thoracic Society (ATS) and the European Respiratory Society (ERS) [19] may further enhance the potential of nNO measurement to be used as a screening test in the clinical setting and especially in countries with limited resources or in areas that lack, or are distant from, PCD-specialist centers [25]. However, the use of a non-perfect screening test such as nNO in isolation may allow for some PCD patients with false negative results to be missed entirely or some non-PCD patients with false-positive results to undergo further diagnostic tests. For this reason, the diagnostic algorithm described as part of Standardized Operating Procedures for PCD diagnosis developed by the EU-funded Seventh Framework Program project BESTCILIA, in 2016, proposed standardized operating procedures for PCD diagnosis and a diagnostic algorithm which recommended that nNO should be performed in parallel with HSVM and confirmatory TEM assessment should follow in case of conflicting results (Additional file 1). Similarly, the recent ERS guidelines for the diagnosis of Primary Ciliary Dyskinesia also recommend a diagnostic algorithm which includes as a first step the parallel performance of both nNO and HSVM and confirmation with TEM in a second step [26]. The rationale of employing a diagnostic algorithm which proposes parallel performance of nNO and HSVM, is to take advantage of the ability of the one test to identify cases that the other test may have missed. Consequently, a positive result in both tests provides evidence that PCD is “highly likely” while a negative result in both tests, especially in the absence of very strong clinical suspicion, provides evidence to consider PCD diagnosis as “extremely unlikely” [26]. Nevertheless, such algorithms require the performance of a significantly higher number of nasal brushings for HSVM and result in higher costs compared to algorithms that only require the performance of a confirmatory test (HSVM or TEM) following a positive screening test.

To better illuminate the decision-making process, the overall diagnostic accuracy of each algorithm, the associated costs as well as the resulting health benefits for PCD patients, need to be addressed and compared. This study aimed to evaluate the diagnostic accuracy, the cost-effectiveness and incremental cost-effectiveness of three distinct diagnostic algorithms for patients referred for PCD diagnostic testing across the European Union through a probabilistic decision analysis framework.

## Methods

### Decision tree model

**Fig. 1:**
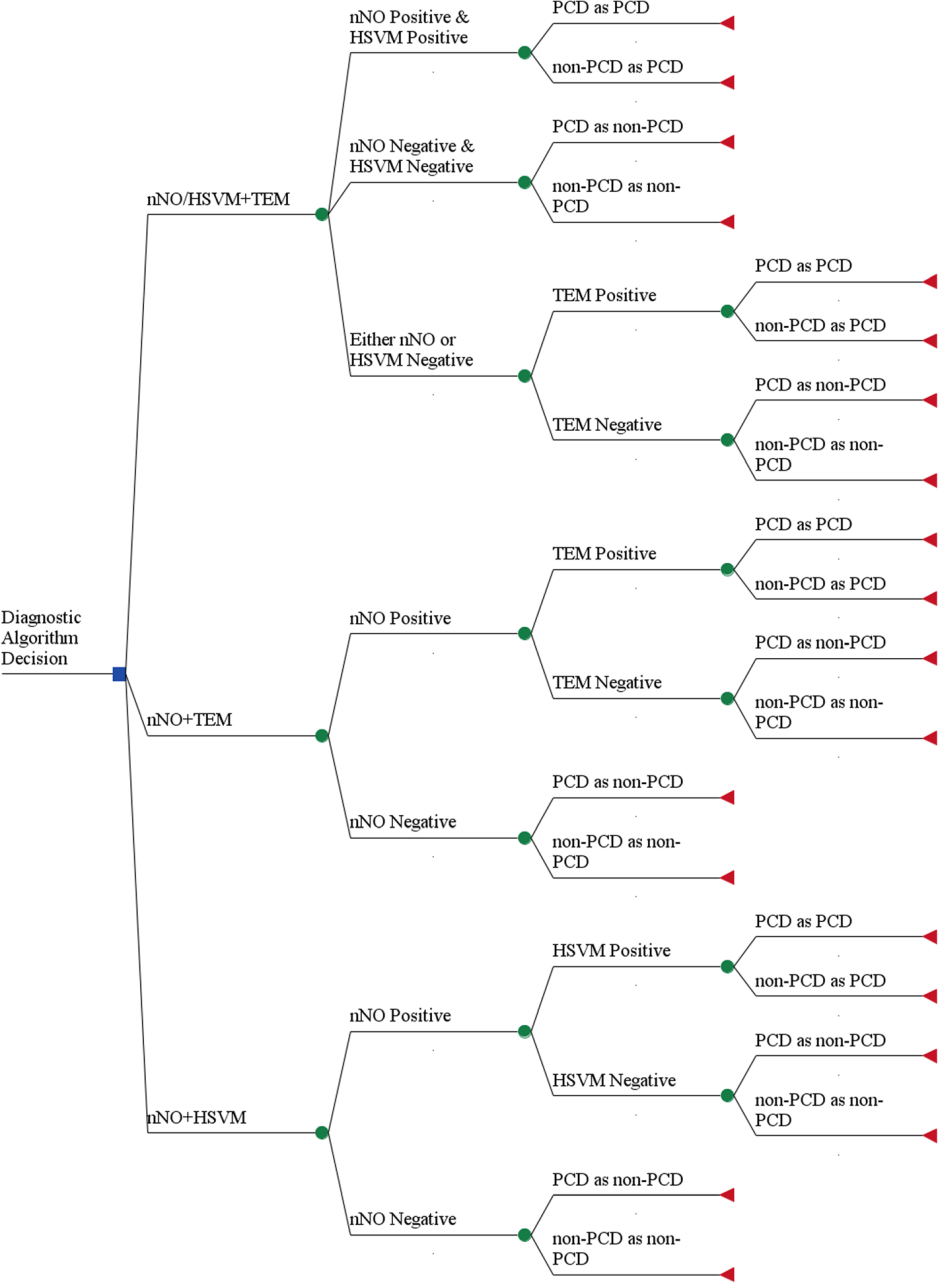
Decision Tree diagram for the three different diagnostic algorithms for PCD. The decision tree begins from the left side and the decision whether to perform nNO + TEM, nNO + HSVM or nNO/HSVM+TEM. Squares represent decision nodes, circles represent chance nodes and triangles represent outcome nodes

Using a probabilistic decision tree model, three diagnostic algorithms were evaluated versus each other and against a baseline of not performing any diagnostic testing for PCD. The three diagnostic algorithms evaluated were a) Sequential testing with nNO screening followed by HSVM only when nNO was positive (nNO + HSVM), b) Sequential testing with nNO screening followed by TEM only when NO was positive (nNO + TEM), c) nNO performed in parallel with HSVM and followed, in cases with conflicting results, by confirmatory TEM (nNO/HSVM+TEM). The decision tree displaying the evaluated three diagnostic algorithms in this study is presented in Fig. 1. The starting population of referrals for PCD diagnosting testing that enters the model was defined as one thousand per year for the whole of the European Union (EU). To estimate the classification of patients under each diagnostic algorithm, Bayes’ Theorem was used. Bayes’ Theorem allows the calculation of probability of suffering from PCD given the pre-test probability of disease and given a positive or negative diagnostic test [27]. The formula for estimating the probability of disease given positive diagnostic test is:

$$ P\left( PCD| Test+\right)=\frac{P\left( Test+| PCD\right)\ast P(PCD)}{P\left( Test+| PCD\right)\ast P(PCD)+P\left( Test+| nonPCD\right)\ast P(nonPCD)} $$

Where P(Test+|PCD) is the probability of positive test given PCD is present (test sensitivity), P(PCD) is the prevalence of PCD in the tested population, P(Test+|nonPCD) is the probability of positive test given disease is not present (1-specificity of the test) and P(non-PCD) is the probability of not having PCD in the tested population. The formula can be rearranged accordingly to calculate probability of PCD given positive diagnostic test, probability of PCD given negative diagnostic test and probability of non-PCD given negative diagnostic test as well as probability of non-PCD given positive diagnostic test. To model the sequence of diagnostic tests in each diagnostic algorithm the resulting probability of PCD given a positive first test as calculated using Bayes’ Theorem was used as the pre-test probability of PCD for the second test. The final modeled health outputs regarding the effectiveness of each diagnostic algorithm included the number of PCD patients confirmed as PCD (True Positive - TP), PCD patients missed (False Negative - FN), non-PCD patients wrongly diagnosed as PCD (False Positive - FP), and non-PCD patients that had a diagnosis of PCD excluded (True Negative - TN). In addition, the annual total cost outcome (in Euros) was calculated for each diagnostic algorithm using a micro-costing approach. This approach involves the recognition of all underlying activities that make up a specific healthcare procedure and the product of resource cost and resource use provides the total cost estimate for the procedure [28]. A detailed description of the diagnostic cost analysis is presented in the Technical Appendix (Additional file 2).

The Incremental Cost-Effectiveness Ratios (ICER) were calculated as the ratio of incremental costs to incremental effectiveness, i.e. [29]:

$$ ICER=\frac{Cost_A-{Cost}_B}{Effect_A-{Effect}_B} $$

Here, Cost_A_ and Cost_B_ are the total annual per-patient costs of performing test algorithms A and B, respectively, and Effect_A_ and Effect_B_ are the number of PCD patients correctly diagnosed with PCD for the same diagnostic algorithms.

The costing perspective of this analysis is societal as it considers all relevant costs for the society (including costs borne by the patient, and/or social services) and not just the costs that are incurred by the healthcare system [30]. Ideally, the cost-effectiveness analysis should not be limited to diagnostic costs and outcomes but should include all expenditures as well as all effectiveness outcomes, preferably in terms of quality-adjusted life years (QALYs), a metric used broadly in the health economics literature [31]. For this reason, a secondary, extended analysis was performed, further described in Additional file 3.

### Model parameter inputs

The prevalence of PCD in the general population was assumed to be 1/15,000 births and the prevalence of PCD among patients referred for diagnostic testing was allocated a probability of 0.32 (95% CI: 0.26–0.39) as reported before [32]. Data regarding the diagnostic accuracy of each test were derived from systematic reviews and meta-analyses, when possible, and from alternative data sources such as large studies and multiple sources when meta-analytic estimates were not available. The parameter inputs for sensitivity and specificity of nNO during Velum Closure (VC) were 0.95 (95% CI: 0.91–0.97) and 0.94 (0.88–0.97) respectively, based on published meta-analytic estimates [33]. For HSVM, the parameter inputs for sensitivity and specificity were 1.0 (95% CI: 0.89–1.00) and 0.92 (95% CI: 0.86–0.96) based on published evidence provided by Boon et al. 2013 and Jackson et al. 2016 [34, 35]. For assessment of ciliary ultrastructure with TEM, the parameter inputs for sensitivity and specificity were 0.74 (95% CI: 0.68–0.80) and 0.91 (95% CI: 0.86–0.96) respectively based on a recent meta-analysis of 11 studies [32]. Sensitivity and specificity values for HSVM and TEM following a positive nNO result were obtained from the study by Jackson et al. 2016 [35]. Table 1 summarizes all parameter values that were part of the basic model.

**Table 1:** Model parameter inputs

Parameter description	Best Estimate (95% CI)	Probability distribution	Source
PCD prevalence among suspect patients	0.32 (0.25–0.39)	Normal (μ: 0.32, SD:0.028)	[32]
Diagnostic Accuracy			
nNO (VC) sensitivity	0.95 (0.91–0.97)	Beta (a: 0.95, b: 0.05)	[33]
nNO (VC) specificity	0.94 (0.88–0.97)	Beta (a: 0.94, b: 0.06)	[33]
TEM sensitivity	0.74 (0.66–0.83)	Beta (a: 0.74, b: 0.26)	[32]
TEM specificity	0.91 (0.86–0.96)	Beta (a: 0.91, b: 0.09)	[32]
HSVM sensitivity	1.00 (0.89–1.00)	Beta (a: 0.99, b: 0.01)	[34, 35]
HSVM specificity	0.92 (0.86–0.96)	Beta (a: 0.92, b: 0.08)	[34]
Diagnostic Costs			
*nNO related cost parameters*)			
nNO Ecomedics CLD88sp (VC) capital cost (€)	40,000 (36,000–44,000)	Gamma (μ: 40,000)	Market Value
nNO Ecomedics CLD88sp (VC) consumables per patient (€)	15 (9–21)	Gamma (μ: 15)	Market Value
nNO operators rate (€/hour)	25 (10–35)	Gamma (μ: 25)	Eurostat
nNO Ecomedics CLD88sp (VC) test duration (hours)	0.5 (0.3–0.7)	Normal (μ: 0.5, SD: 0.1)	Based on ATS/ERS [19]
nNO Ecomedics CLD88sp equipment lifespan (years)	15 (13–17)	Normal (μ: 15, SD: 1)	Market Value
nNO Ecomedics CLD88sp (VC) annual maintenance (€)	1300 (1100–1500)	Gamma (μ: 1300)	Market Value
*HSVM related cost parameters*			
Capital cost HSVM – SAVA system (€)	5000 (3000–7000)	Gamma (μ: 5000)	Market Value (incl. Camera and software)
HSVM consumables (€)	30 (26–34)	Gamma (μ: 30)	Market Value
HSVM operators rate (€/hour)	25 (10–35)	Gamma (μ: 25)	Eurostat
HSVM equipment lifespan (years)	15 (10–20)	Normal (μ: 15, SD: 2)	Assumption
HSVM test duration (hours)	2 (1.6–2.4)	Normal (μ: 2, SD: 0.2)	Based on Sisson J 2003 [17]
*TEM related cost parameters*			
TEM capital cost (€)	100,000 (90,000–110,000)	Gamma (μ:100,000)	Market Value
TEM consumables (€)	120 (90–140)	Gamma (μ:120)	Market Value
Brushing Time (hours)	0.2 (−)	Constant: (Brushing Time: 0.2)	Assumption
TEM operators rate (€/hour)	25 (10–35)	Gamma (μ: 25)	Eurostat
TEM test duration (hours)	10 (6–18)	LogNormal (Median: 10, gsd: 1.3)	[12, 35]
TEM equipment lifespan (years)	30 (20–40)	Normal (μ: 30, SD: 5)	Assumption
Physician’s rate (€/hour)	50 (30–70)	Gamma (μ: 50)	Eurostat
TEM annual maintenance (€)	2000 (1300–2600)	Gamma (μ: 2000)	Assumption

### Characterization of uncertainty

**Fig. 2:**
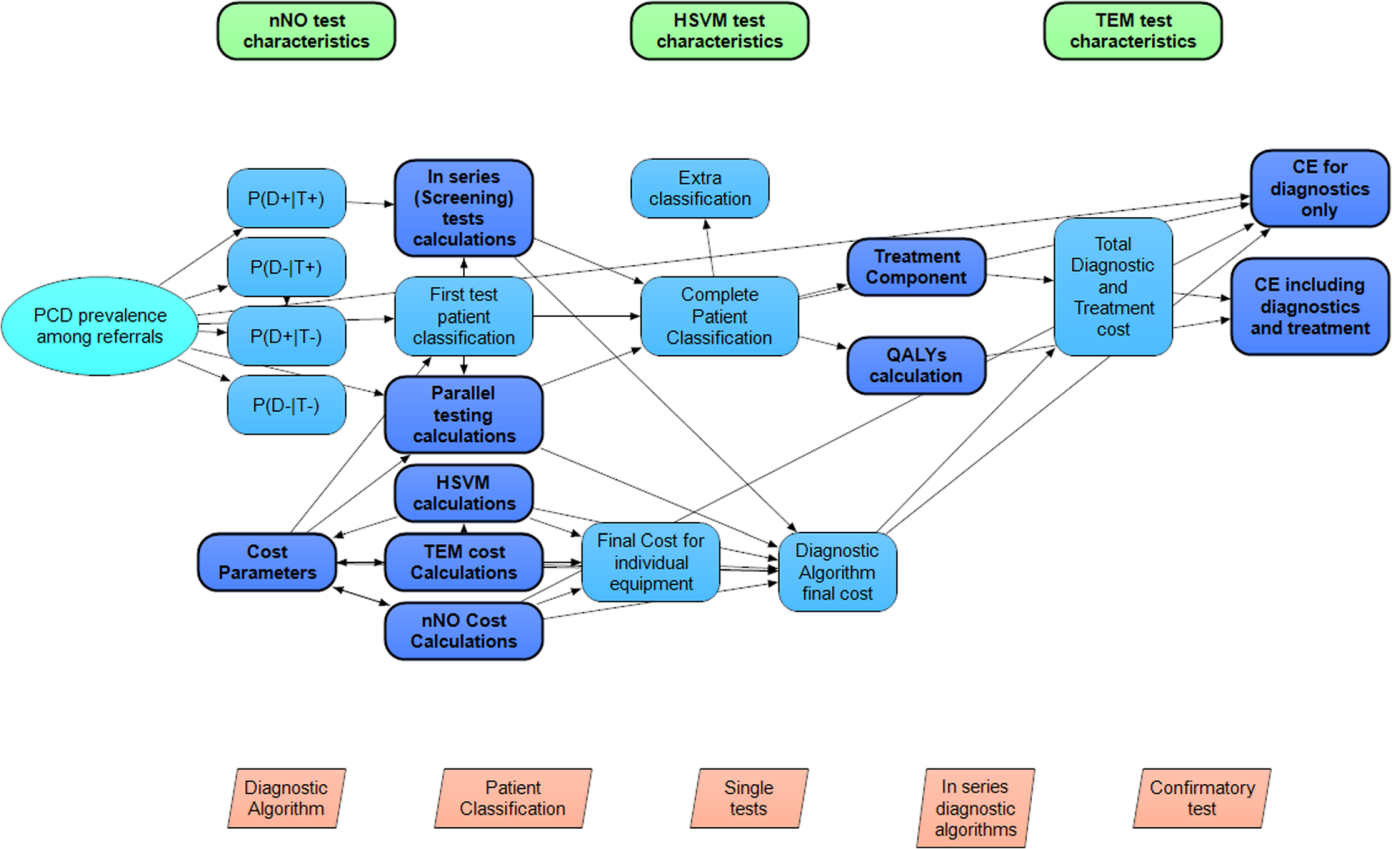
Model Overview. Schematic Overview of ANALYTICA model

Reported uncertainty around pooled estimates of the meta-analyses of diagnostic effectiveness and uncertainties about the true value of costs and other parameters are reflected by the probability distributions around the parameter means which are used in this model. A Cost-Effectiveness Acceptability Curve was used to demonstrate the uncertainty in the estimation of the ICER [36] while the robustness of the estimated ICER was tested through the performance of one-way sensitivity analyses where the input parameters varied over their range. All parameters and equations constitute the final model which was developed with ANALYTICA 101 edition (Lumina decision systems, CA, United States). The model was executed with 3000 iterations per “model run” using Latin Hypercube sampling to generate samples from the underlying parameter probability distributions. The model can be assessed online (Additional file 4) and a model overview is presented in Fig. 2.

## Results

**Fig. 3:**
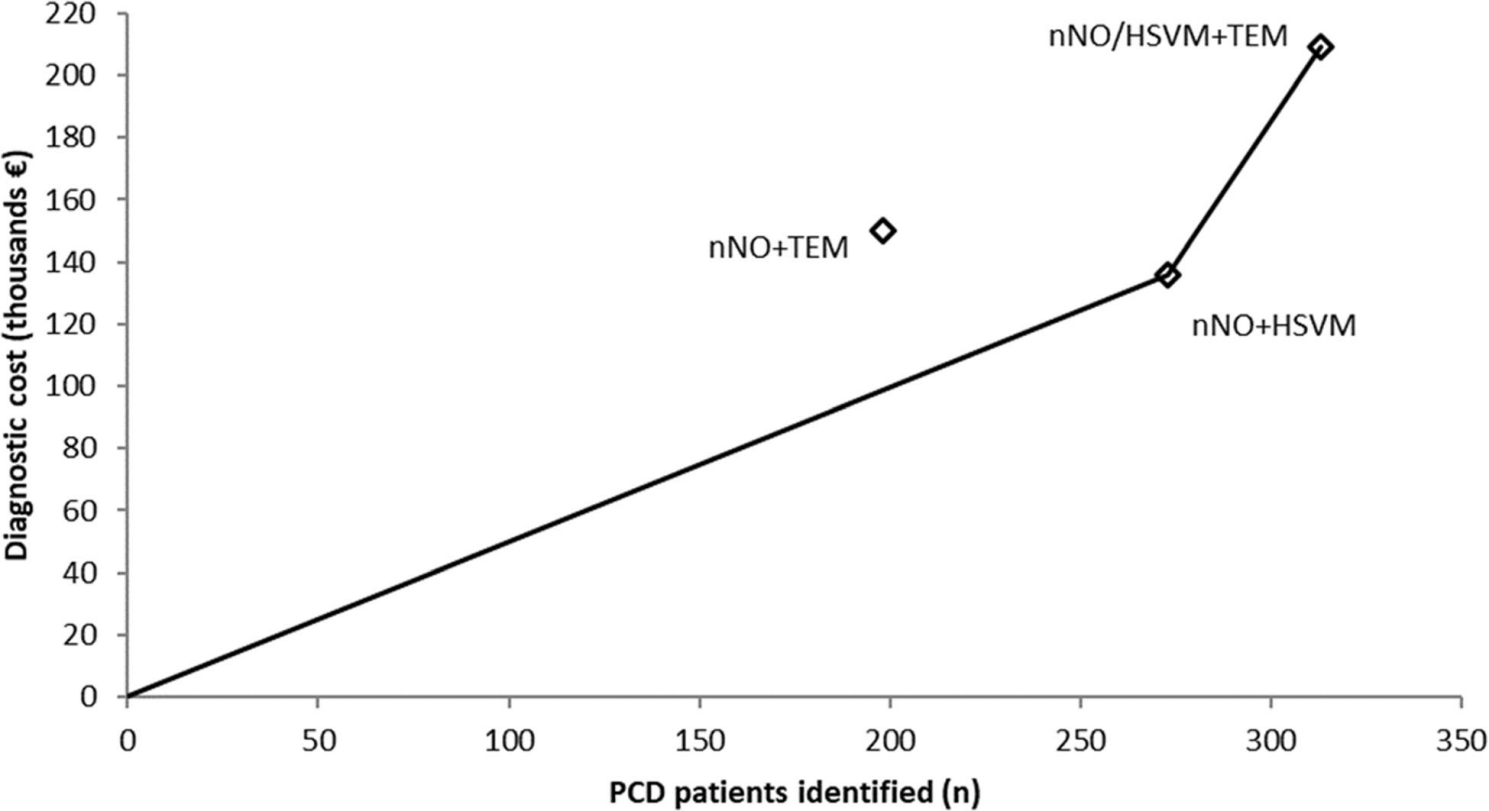
Cost-effectiveness frontier for the three different diagnostic algorithms for PCD. Diagnostic algorithms nNO + HSVM and nNO/HSVM+TEM are cost-effective alternatives at different WTP thresholds. Diagnostic algorithm nNO + TEM is dominated by nNO + HSVM

**Fig. 4:**
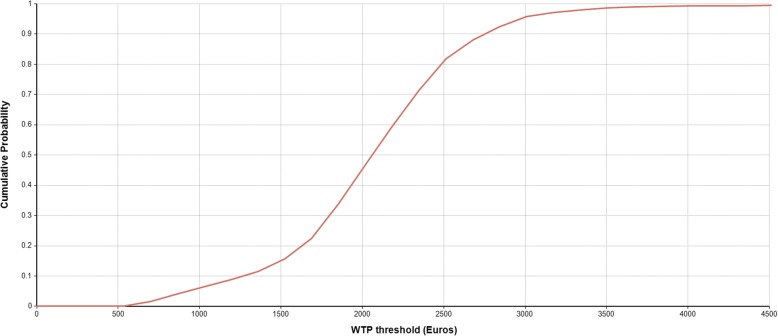
Cost Effectiveness Acceptability Curve for nNO/HSVM+TEM. The probability that diagnostic algorithm nNO/HSVM+TEM is cost-effective for a range of WTP thresholds

**Fig. 5:**
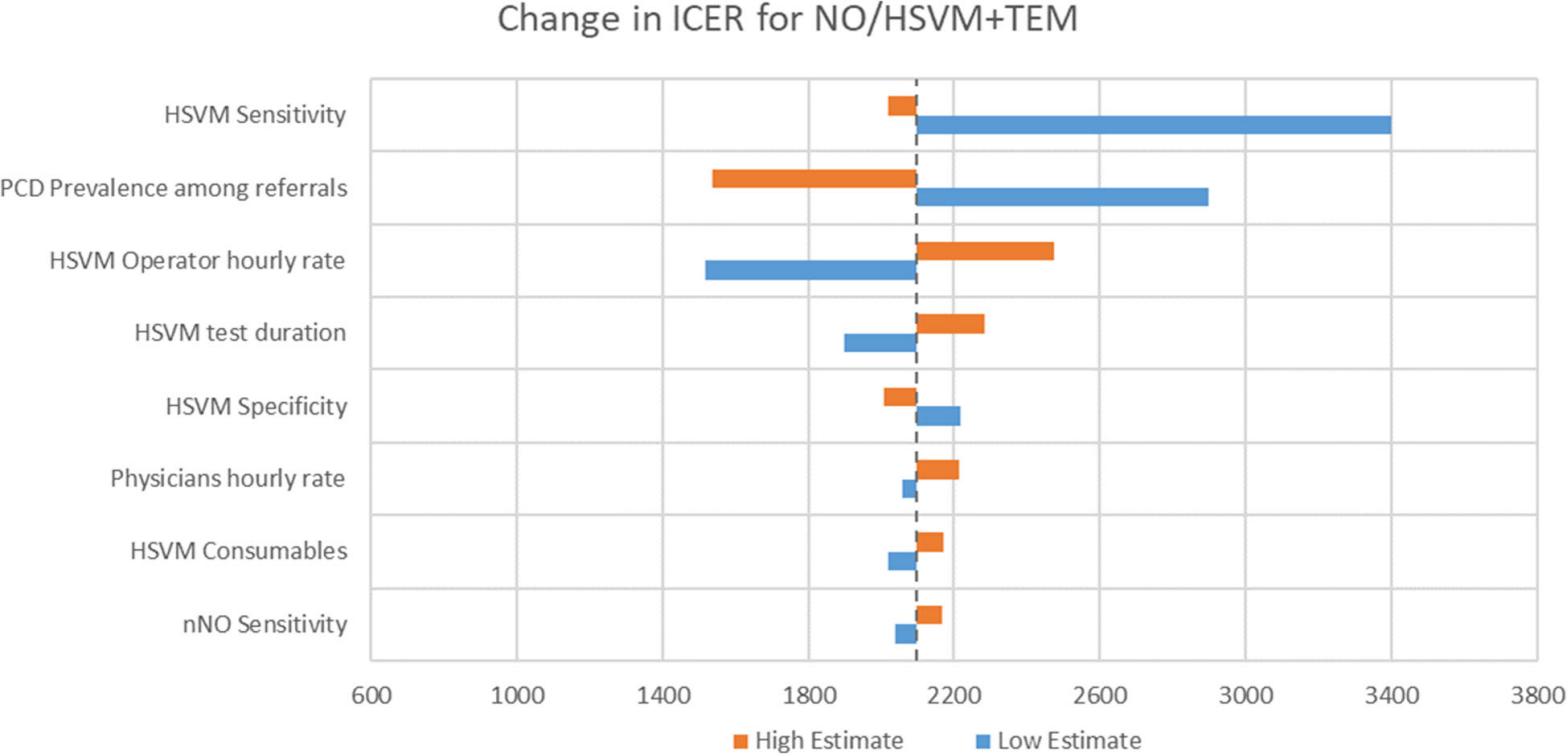
One-way sensitivity analyses for ICER. Tornado diagram demonstrating one-way sensitivity analyses of modelled parameters that affect the ICER. The dashed vertical black line represents the base case value (ICER = 2097 Euros/additional PCD case identified). PCD: Primary Ciliary Dyskinesia, nNO: nasal Nitric Oxide, HSVM = High Speed Video Microscopy, ICER = incremental cost-effectiveness ratio.Cost Effectiveness

The model output for TP, FN, TN and FP and estimates of net sensitivity, net specificity, net positive predictive value and net negative predictive value for the application of each diagnostic algorithm in a hypothetical cohort of 1000 patients suspected of PCD is presented in Table 2. Table 3 compares mean diagnostic costs with the number of PCD cases identified and reports relevant CERs and ICERs. Deterministic comparison for mean costs and effects demonstrated that the nNO/HSVM+TEM was the most effective algorithm but also the costliest (313 PCD cases identified/year, 209 thousand €/year). nNO + HSVM was the second most effective (273 PCD cases identified/year, 136 thousand €/year) while nNO + TEM was the least effective (198 PCD cases identified/year, 150 thousand €/year). The most cost-effective algorithm was nNO + HSVM with a CER of €653/PCD case identified, followed by nNO/HSVM+TEM (€678/PCD case identified) and nNO + TEM (€975/PCD case identified). The cost effectiveness frontier in presented in Fig. 3 and the resulting ICER for nNO/HSVM+TEM compared to nNO + HSVM, the second most effective algorithm, is €2097 per additional PCD case identified. The nNO + TEM algorithm is dominated (simple dominance) by nNO + HSVM as it is more expensive but less effective compared to nNO + HSVM. Figure 4 presents the cost-effectiveness acceptability curve (CEAC) for nNO/HSVM+TEM. The CEAC demonstrates the uncertainty in the estimation of ICER and provides information about the probability of nNO/HSVM+TEM being more cost effective compared to nNO + HSVM for a range of potential monetary amounts (termed willingness to pay (WTP) thresholds) that a decision maker might be willing to pay to correctly diagnose an additional PCD case. For a WTP threshold equal to €2500 the probability of nNO/HSVM+TEM being cost effective is over 70% and for a WTP threshold equal to €3500 the probability is over 97%. The results of one-way sensitivity analyses demonstrated that the modelled mean ICER for nNO/HSVM+TEM is primarily affected by changes in the input value for HSVM sensitivity, followed by the changes in input values for the prevalence of PCD among suspect patients. Changes in the input values of other modelled parameters had smaller effects on the ICER (Fig. 5). Results of secondary analysis are presented in Additional file 3.

**Table 2:** Diagnostic accuracy of nNO + TEM, nNO + HSVM and nNO/HSVM+TEM algorithms

Classification	Diagnostic Algorithm		
	NO+TEM	NO+HSVM	NO/HSVM+TEM
PCD as PCD (% of PCD)	198 (62%)	273 (85%)	313 (98%)
PCD as non-PCD (% of PCD)	122 (38%)	47 (15%)	7 (2%)
Non-PCD as non-PCD (% of non-PCD)	678 (99.7%)	680 (100%)	674 (99%)
Non-PCD as PCD (% of non-PCD)	2 (0.003%)	0 (0%)	6 (1%)
Net Sensitivity	62%	85%	98%
Net Specificity	99.7%	100%	99%
Net PPV	99%	100%	98%
Net NPV	85%	94%	99%

*PPV* Positive Predictive Value, *NPV* Negative Predictive Value

**Table 3:** Diagnostic costs per year, identified PCD cases per year (mean and 95% Confidence Interval)

Diagnostic Algorithm	Diagnostic Cost per annum in thousand €	PCD cases identified per annum	ICER (€/PCD case identified)	
			Compared to No screening	Compared to next most effective algorithm^a^
Do nothing	0	0	-	-
NO + HSVM	136 (109–177)	273 (105–335)	653 (385–1110)	653 (385–1110)
NO + TEM	150 (118–208)	198 (76–242)	975 (595–1605)	Dominated
NO/HSVM + TEM	209 (173–261)	313 (231–373)	678 (508–1003)	2097 (770–3233)

^a^ICER compared to next less expensive algorithm omits from consideration those algorithms that are “dominated” (make health worse and cost more). Hence, we compare NO/HSVM+TEM (last row) to NO+HSVM (2nd row) and not to NO+TEM (3rd row) because NO+TEM is dominated (it costs more than NO+HSVM but identifies fewer cases)

## Discussion

The high genetic heterogeneity that characterizes PCD and the resulting inability to rely on a single test to confirm or exclude diagnosis of the disease has led to increased research interest in specialized diagnostic testing for PCD in recent years. This study compares three diagnostic strategies currently in use for diagnosing PCD and reports on their effectiveness and cost-effectiveness under a societal costing perspective. Data were drawn primarily from meta-analyses of diagnostic effectiveness or published estimates from large studies and were synthesized in a probabilistic cost effectiveness model.

The results presented here demonstrate that when the effectiveness outcome is defined as the number of PCD patients identified, nNO/HSVM+TEM is the most effective diagnostic algorithm followed closely by nNO + HSVM. Both nNO/HSVM+TEM and nNO + HSVM are significantly more effective compared to the third diagnostic strategy evaluated, nNO + TEM. Mean estimates of CERs demonstrate that nNO + HSVM was the most cost-effective option and a decision maker should expect to pay on average an amount equal to €2097 per additional case identified if nNO/HSVM+TEM is implemented. Whether the effectiveness outcome is defined as the number of PCD patients identified or as the number of QALYs saved nNO/HSVM+TEM was still the most effective algorithm followed by nNO + HSVM and nNO + TEM. Nevertheless, the results of the extended model, which are expressed in Euros per QALY saved, demonstrate that all three diagnostic algorithms appear to be very cost-effective. Compared to no screening, the cost per QALY gained for the three diagnostic algorithms examined here ranged from €6674 to €12,930, an estimate which is lower than WTP thresholds commonly used by regulatory authorities around the world. Such WTP thresholds range between £20,000 and £30,000 per QALY saved in the UK [37] or the more conventional WTP threshold of $50,000 per QALY saved, commonly used in the US [38] or even more recently suggested WTP thresholds in the range of $100,000 to $200,000 per QALY [39].

Diagnostic algorithms including nNO measurement during VC as an initial screening could be cost-effective. However, our results demonstrate that nNO screening is more effective when the confirmatory test is HSVM and not TEM. Although in the past TEM was considered the gold standard [13], it is now known to miss an important fraction of PCD patients [32], mainly those with biallelic mutations in *DNAH11* gene [40] and those with specific ultrastructural abnormalities (nexin link defects) that are not easily detectable by standard TEM [41]. Furthermore, it requires access to a specialized lab with personnel experienced in staining and interpretation of TEM micrographs and consequently involves considerable resource allocation [42]. At the same time, TEM studies are usually time consuming and results are frequently obtained and communicated to patients considerably later than results of other tests thus contributing to patient distress [43]. HSVM is easier, considerably faster and cheaper than TEM as it is usually performed on the same day following nasal brushing and the equipment required consists of standard microscope, a high speed video camera and a standard computer loaded with specialized software. It has also been reported to be a highly sensitive and specific test [35] thus it significantly outperforms TEM as a confirmatory test both in terms of overall effectiveness and cost. However, extra caution is required with HSVM as it may be affected by observer subjectivity and non-PCD specific findings which may interfere with the motility interpretation [22]. Overall, the parallel performance of two highly specific and sensitive tests such as nNO and HSVM during the first step of the diagnostic algorithm, followed by confirmatory TEM in only the few cases of conflicting findings, results in the identification of most PCD patients and does not require the performance of the more expensive and time consuming TEM analysis for the largest part of the cohort of suspect patients.

In this study we did not include diagnostic algorithms that included immunofluorescence (IF) and/or genetic testing for PCD. Although a recent study has reported the first diagnostic accuracy and cost estimates for immunofluorescence testing in PCD [44], the use of this test is still very limited (as it is performed only in a small number of few highly specialized centers around the world). Genetic testing, on the other hand, is available in many centers around the world. However, as yet, there is little standardization of procedures for the conduct and interpretation of results. Different centers may use different technologies and may not test for the same number of genes [45, 46]. Thus estimation of the effectiveness or the cost of genetic testing as diagnostic for PCD was not possible at this stage and it was not included in the diagnostic algorithms considered in our analysis. This approach is in line with the recent guidelines published by the ERS where genetic testing was recommended as a last step following abnormal TEM primarily for further characterization of the underlying defect or as a final diagnostic test if all other tests were inconclusive. For immunofluorescence there was no ERS recommendation towards its use as a diagnostic test given the scarcity of evidence [26].

The main strength of this study is that it makes use of evidence-based estimates and individual good quality studies on the diagnostic accuracy of nNO, TEM and HSVM and the prevalence of PCD among cohorts of referred suspect patients. With the use of Bayes’ Theorem, it was possible to estimate the diagnostic effectiveness of sequential tests and to compare the effectiveness of diagnostic algorithms instead of simply comparing the effectiveness of isolated tests, as had been done in the past. In addition, our analysis of the costs involved in diagnostic testing followed standard approaches for economic analysis of healthcare procedures [28] and made use of the extensive literature on the effort, equipment and consumables involved in the performance of nNO [47, 48], HSVM [13, 35] and TEM [18]. Based on this evidence, we were able to calculate effectiveness and economic outcomes (number of PCD patients identified, total diagnostic costs) as well as robust CERs, ICERs and identify the cost-effectiveness frontier.

Nonetheless, this study also has some limitations. In the main analysis, although our data on diagnostic accuracy are mostly based on meta-analyses of well conducted studies, these are characterized by a degree of heterogeneity [32, 33]. On the other hand, our data on diagnostic cost parameters are primarily based on realistic estimates of current market values, although these may not be uniform across all EU countries. The one-way sensitivity analyses for the diagnostic ICER for NO/HSVM+TEM demonstrates that our results are most sensitive to variations in HSVM sensitivity and PCD prevalence among suspect patients. A recent, large study on diagnostic accuracy of HSVM reported a sensitivity of 100%, which is in line with the value used in our model [49]. Nevertheless, it is possible that PCD prevalence among referred suspect patients varies considerably between countries, as different countries may utilize different diagnostic protocols and referral patterns [20, 50, 51]. Even so, these disparities between countries are expected to be reduced in the future with the increasing use of clinical scoring tools [52], the intercalation between PCD clinicians in international networking projects such as the BEAT-PCD COST project [53] and the establishment of European Reference Networks for rare diseases including PCD (ERN-LUNG) [54].

Most limitations of this work however, relate to the considerable uncertainty of the parameters used in the secondary analysis and for this reason the results of the basic and extended model are presented separately. As a result, caution is advised before generalizing the results of this study, especially those regarding the extended model. Another limitation of the extended model is that despite empirical evidence about various approaches for the treatment of PCD, at the moment there are no widely recognized PCD-specific treatment protocols. The efficacy of a few treatment approaches are now under investigation through randomized control trials, for example, those now underway on the effect of azithromycin for antibiotic prophylaxis [55]. Furthermore, there are no published estimates of the annual (or lifetime) cost of various options for treatment of PCD. Although we used credible sources to estimate patient associated cost [56] and cost of each procedure (resource cost) [57–59], we had to rely on our own experience with the disease to characterize the typical frequency of treatment (resource use). To address this limitation, the underlying uncertainty in each parameter was characterized and included in the model. Through Latin Hypercube sampling and Monte Carlo analysis, these uncertainties in individual parameters were propagated through the model and are reflected in the uncertainty in final model outputs.

Evidence about treatment costs is especially weak. We found no evidence of the cost of treatment of PCD patients who remain undiagnosed; and only limited evidence about the cost of treatment of PCD patients who are properly diagnosed. A sensitivity analysis was conducted to determine whether differences in the overall costs of treatment of diagnosed and undiagnosed PCD patients affected the estimates of cost-effectiveness from the extended model. The overall order of diagnostic algorithms was not affected and nNO/HSVM+TEM was the most cost efficient algorithm in all scenarios. However, the magnitude of the difference in the cost effectiveness of the three algorithms was significantly affected, with nNO/HSVM+TEM becoming relatively more cost-effective when it was assumed that the cost of treating undiagnosed PCD patients was at least 3 times greater than the treatment cost for properly-diagnosed PCD patients. This highlights the importance of future studies which address the economic cost of treatment in PCD patients before and after diagnosis.

We encountered a similar lack of data on the impact of PCD on life expectancy and the patients’ valuation of health status (health utility). Currently, PCD is considered a disease characterized by normal or near-normal life span, although cases of premature mortality among PCD patients are reported in the literature [8, 60]. To date, no study has reported on patients’ life expectancy and this lack of information could be attributed to the fact that PCD has been studied primarily in small cohorts in the pediatric setting. The recently established prospective international PCD registry [61], which now includes several thousands of pediatric and adult patients, is expected in the next few years to provide data on disease progression and life-expectancy. Likewise, to date no study has reported on health state utilities in PCD and thus we used in our calculations data on health utilities from mild Cystic Fibrosis patients that have been previously reported to have similar clinical severity with PCD [62]. The one-way sensitivity analyses in the extended model, which included treatment costs and outcomes, demonstrated that the most important parameter impacting the CER of nNO/HSVM+TEM was PCD health utility followed by loss of productivity, reduction in life expectancy and antibiotics cost. In order to further improve our understanding of the disease and better inform the development and improvement of guidelines for PCD diagnosis and treatment, future studies aiming to assess the real value of cost-of-illness, healthcare utilization estimates and health state utilities are urgently needed.

## Conclusions

Across the world, many PCD diagnostic centers follow a variety of algorithms for diagnosing PCD and, most likely, in some low income countries, there is a complete lack of specialized diagnostic testing. The results of this study suggest that a diagnostic algorithm which includes nNO during VC as a screening test followed by confirmatory HSVM identifies approximately 85% of PCD patients with a mean CER of €653per PCD case identified. The algorithm which maximizes the number of PCD patients identified involves parallel performance of nNO and HSVM as the first step, followed by TEM as a confirmatory test for the few cases where nNO and HSVM yield conflicting results, with a corresponding ICER of €2097 per additional PCD patient identified. Decision analysis methods and the evidence from this study can inform the dialogue on evidence-based guidelines for PCD diagnostic testing. Future studies in understudied aspects of PCD relating to quality of life, treatment efficiency and associated costs are urgently needed to help the better implementation of these guidelines across various healthcare systems.

## Additional files

Additional file 1: BESTCILIA Diagnostic Algorithm for PCD. (PDF 188 kb)

Additional file 2: Technical Appendix. (DOCX 20 kb)

Additional file 3: Secondary Analysis. (DOCX 169 kb)

Additional file 4: Cost-effectiveness ANALYTICA model. (ANA 51 kb)

## Data Availability

The datasets used and analyzed during the current study are available from the corresponding author on reasonable request.

## Abbreviations

ATS: American Thoracic Society
CEAC: Cost Effectiveness Acceptability Curve
CER: Cost Effectiveness Ratio
ERN: European Reference Networks
ERS: European Respiratory Society
EU: European Union
HSVM: High Speed Video Microscopy
ICER: Incremental Cost Effectiveness Ratio
nNO: Nasal Nitric Oxide
PCD: Primary Ciliary Dyskinesia
QALY: Quality Adjusted Life Years
TEM: Transmission Electron Microscopy
VC: Vellum Closure
WTP: Willingness to pay
