# Supplementary material for: Cost-effectiveness analysis of three algorithms for diagnosing primary ciliary dyskinesia: a simulation study
Source: Orphanet J Rare Dis. 2019 Jun 13;14:142. doi: 10.1186/s13023-019-1116-3 (PMC6567920; doi:10.1186/s13023-019-1116-3)
Supplement: Supplementary file 1 — BESTCILIA Diagnostic Algorithm for PCD. (PDF 188 kb) [file 13023_2019_1116_MOESM1_ESM.pdf]

Diagnostic Approach & Algorithm for PCD

Main manifestations of PCD in the antenatal, neonatal, childhood and adolescence-adulthood periods.

Antenatal

Situs inversus totalis or heterotaxy on antenatal ultrasound scanning;
25% of individuals with situs inversus totalis have PCD. The prevalence of PCD within the heterotaxic subclass is unknown.
40–50% of PCD patients present with situs inversus totalis (Kartagener's syndrome in PCD.
6% show heterotaxy (situs ambiguus)
Mild fetal cerebral ventriculomegaly

Neonatal

75% of full-term neonates with PCD exhibit neonatal respiratory distress requiring supplemental oxygen for days to weeks.
Continuous rhinorrhoea from the first days of life.
Mirror-image organ arrangement and other forms of heterotaxy (see Antenatal rows above)
Hydrocephalus may occur in some individuals with PCD, and may reflect dysfunctional ependymal cilia

Childhood

Chronic productive or wet-sounding cough, associated or not with recurrent atelectasis or pneumonia
Atypical asthma that is nonresponsive to treatment, especially if a wet-sounding cough is present
Idiopathic bronchiectasis
Daily rhinitis without remission; nasal polyps are rare at this age
Severe chronic sinusitis in older children
Otitis media with effusion
Hearing loss

Adolescence and adult life (Same as for childhood)

Bronchiectasis more evident in adulthood (83%)
Chronic mucopurulent sputum production is common
Digital clubbing may also be found.
Pulmonary function tests usually show a progressive obstructive or mixed pattern.
Nasal polyposis and halitosis
Infertility in males (50%) due to immotility of spermatozoa. "

Indications for Referral and Testing

1) Children who are found at any age to have situs inversus totalis, or any heterotaxic syndrome, should have PCD excluded by appropriate tests.
Level of evidence: high; benefit: substantial; strength of recommendation: strong.

2) Children who are found to have cerebral ventriculomegaly at any age, in the absence of an obvious cause, and, in particular, if there are other features suggestive of PCD, should

probably have this diagnosis excluded.
Level of evidence: low; benefit: small; strength of recommendation: weak.

3) Siblings of probands should have PCD excluded.
Level of evidence: high; benefit: substantial; strength of recommendation: strong.

4) Babies with otherwise unexplained neonatal respiratory distress, particularly if there are other features of PCD, should have this diagnosis excluded.
Level of evidence: high; benefit: substantial; strength of recommendation: strong.

5) Children with chronic productive cough, bronchiectasis of unknown cause or severe upper airway disease should be considered for diagnostic testing for PCD after other more common diagnoses have been excluded, particularly if there are other features of PCD.
Level of evidence: expert opinion; benefit: substantial; strength of recommendation: strong.

6) Males with immotile sperm should be referred for diagnostic testing for PCD.
Level of evidence: high; benefit: substantial; strength of recommendation: strong.

7) Females with recurrent ectopic pregnancy should be considered for referral for diagnostic testing, especially if there are other features of PCD.
Level of evidence: low; benefit: small; strength of recommendation: weak."

Algorithm for performance of diagnostic tests

Patient selection criteria:

A. All patients with the above indications (1, 2, 3, 4, 5, 6 and 7) should be invited for nasal NO testing and high-speed video-microscopy.
B. In any patient if either nasal NO or HSVM is abnormal proceed with TEM and IF performance.
C. In patients with very strong clinical suspicion (SI and bronchiectasis [Kartagener syndrome] or neonatal respiratory distress + classical PCD history) and normal nasal NO and HSVM: TEM and IF are also performed (possibly we here identify subtle defects)
D. In any patient if diagnosis solely relies on HSVM findings only we perform this test at least (3x). If on three occasions an identical beating defect is identified, the diagnosis is confirmed.
