# Supplementary material for: Cost-effectiveness analysis of three algorithms for diagnosing primary ciliary dyskinesia: a simulation study
Source: Orphanet J Rare Dis. 2019 Jun 13;14:142. doi: 10.1186/s13023-019-1116-3 (PMC6567920; doi:10.1186/s13023-019-1116-3)
Supplement: Supplementary file 2 — Technical Appendix. (DOCX 20 kb) [file 13023_2019_1116_MOESM2_ESM.docx]

**Technical Appendix**

**For**

**Cost-effectiveness Analysis of Three Algorithms for Diagnosing Primary Ciliary Dyskinesia**

**Diagnostic Cost Analysis:**

The analysis was conducted from the perspective of an average European Union (EU) Healthcare System and thus it is confined to direct medical costs based on EU rates.

The amortized annual cost estimates for the various pieces of diagnostic equipment (nNO analyzer, HSVM system and TEM) were calculated by the formula:

$$M=P*i*\frac{{(1+i)}^{N-1}}{({1+i)}^{N}-1}$$

Where P is the capital cost, i is the annual discount rate (treated as 0.05 per year in this analysis) and N is the useful life (lifespan) of the equipment. Amortized annual cost was added to the annual operating and maintenance costs (including labor costs, consumables and maintenance) to estimate the total annual diagnostic cost for each diagnostic algorithm.

The annual total operating and maintenance cost for a hypothetical cohort of 1000 suspect patients referred for specialised PCD testing was calculated using the micro-costing approach which involved the following steps:

1. Identification of all cost generating procedures (recourses) involved in diagnosting testing for PCD
2. Estimate unit resource cost based on average EU prices for different resources.
3. Estimate the resource usage based on model output.
4. Combine the information on unit recourse costs with resource usage and sum across all identified resources as described by the general formula:

$$\sum_{i=1}^{k} {RC}_{i}+{RU}_{i}$$

Where *k* refers to the total number of resources involved in PCD diagnosting testing and *RC_i_* and *RU_i_* refer to the resource cost and resource use estimated for resource *i*.

**Direct medical costs included in PCD diagnostic testing**

Direct medical costs include the cost of nasal Nitric Oxide (nNO) measurement, the physician office visit cost for the performance of nasal brushing and the laboratory cost involved in the performance of the High Speed Video Microscopy (HSVM) and Transmission Electron Microscopy (TEM) analyses on the acquired sample. The cost of nNO, HSVM and TEM equipment purchase, disposables, labour and maintenance constitute the direct medical cost and the detailed breakdown of these diagnostic procedures into separate resources and their pricing is described in Table 1.

**Calculation of diagnostic costs:**

The electron microscope used for TEM analysis and the analyser used for NO measurement are not only used for PCD diagnosis but could be used for other applications as well. On the contrary HSVM is expected to solemnly be utilised for PCD diagnosis. As a result, two slightly different approaches are followed for the calculation of diagnostic costs for HSVM and nNO and TEM. More specifically, in the case of nNO and TEM the final cost of the diagnostic procedure calculated across all resources (e.g. capital cost, labour, consumables etc) has to be corrected using the ratio of equipment use to the total equipment efficiency which is calculated as follows:

$$\frac{Number of patients tested*T_{Test}}{{Efficiency}_{Equipment}}$$

Where T_Test_ refers to the duration of the test in hours and Efficiency_Equipment_ refers to the total time (hours) that the equipment could theoretically be used over a year.

**Diagnostic cost (HSVM):**

The operational cost per patient for each diagnostic test was calculated using the formula:

$${OC}_{per patient}=\left[ T_{Test} \left( hours \right)*CR (\frac{€}{hour}) \right]+{Consumables}_{per patient}$$

Where *OC_per patient_* refers to Operational Cost per patient, *T_Test_* refers to the duration of the test in hours and *CR* refers to Compensation Rate for the diagnostician in €/hour. The total annual operational cost (TOC) was calculated as follows:

$$TOC={OC}_{per patient}*Number of patients tested$$

Total annual Operational and Maintenance Cost (TOCM) which includes the operational cost as well as annual payments towards the capital investment and annual maintenance is calculated as:

$$TOCM=TOC+AC+AM$$

Where *TOC* refers to Total Operational Cost, *AC* refers to amortized cost and *AM* refers to expected annual maintenance of the equipment.

**Diagnostic cost (nNO and TEM):**

The operational cost per patient for each diagnostic test was calculated using the formula:

$${OC}_{per patient}=\left[ T_{Test} \left( hours \right)*CR (\frac{€}{hour}) \right]+{Consumables}_{per patient}$$

Where *OC_per patient_* refers to Operational Cost per patient, *T_Test_* refers to the duration of the test in hours and *CR* refers to Compensation Rate for the diagnostician in €/hour. The total annual operational cost (TOC) was calculated as follows:

$$TOC={OC}_{per patient}*{Efficiency}_{Equipment}$$

Total annual Operational and Maintenance Cost (TOCM) which includes the operational cost as well as annual payments towards the capital investment and annual maintenance is calculated as:

$$TOCM=TOC+AC+AM$$

For nNO measurement:

$$\left[ \frac{Number of patients tested*T_{Test}}{{Efficiency}_{Equipment}} \right]*TOCM$$

For TEM analysis (also includes sample processing cost (PC) prior TEM analysis):

$$\left( Number of patients tested*PC \right)+\left[ \frac{Number of patients tested*T_{Test}}{{Efficiency}_{Equipment}} \right]*TOCM$$

**Patient-associated costs:**

For the performance of PCD diagnostic testing, the patient is required to visit a PCD referral centre and all procedures that involve the patient (including registration, paperwork, clinical examination, nasal nitric oxide measurement and nasal brushing) are completed in a few hours. As a result, the main costs that are borne by the patient are transportation cost and loss of productivity for the time spend at the PCD referral centre. The total patient transportation costs were calculated as:

$$Patients tested*travel distance to and from PCD center \left( km \right)*travel cost per km$$

Given that in many countries, CF referral centres are also referral centres for PCD, the travel distance value was based on a previous analysis that reported the travel distance to a CF reference centre as approximately 100km (Johnson B et al., 2018). Travel cost per km in the EU was modelled as 0.19 Euros per km (Kanters TA et al., 2017). Loss of productivity was calculated using the human capital approach (Krol M et al., 2014) and assumes that patient and/or caregivers would spend half working day off work:

$$Patients tested*time at PCD referal center \left( hrs \right)*EU hourly labour cost$$

The mean EU hourly labour cost based on 2018 data equaling to 27.4 Euros was used: (<https://ec.europa.eu/eurostat/statistics-explained/index.php/Wages_and_labour_costs)>.

References:

1. Johnson B, Ngueyep R, Schechter MS, Serban N, Swann J. **Does distance to a cystic fibrosis center impact health outcomes?** 2018 Pediatric pulmonology, 53(3), 284-292.
2. Kanters TA, Bouwmans, CA, van der Linden N, Tan SS, Hakkaart-van Roijen L. **Update of the Dutch manual for costing studies in health care.** 2017, PloS one, 12(11), e0187477.
3. Krol M, Brouwer W (2014). **How to estimate productivity costs in economic evaluations.** 2014 Pharmacoeconomics, *32*(4), 335-344.
