# Supplementary material for: Cost-effectiveness analysis of three algorithms for diagnosing primary ciliary dyskinesia: a simulation study
Source: Orphanet J Rare Dis. 2019 Jun 13;14:142. doi: 10.1186/s13023-019-1116-3 (PMC6567920; doi:10.1186/s13023-019-1116-3)
Supplement: Supplementary file 3 — Secondary Analysis. (DOCX 169 kb) [file 13023_2019_1116_MOESM3_ESM.docx]

**Secondary Analysis**

**Methods Employed in secondary Analysis**

The secondary analysis aimed to (1) broaden our characterization of costs to include all healthcare expenditures, and (2) quantify effectiveness in terms of quality adjusted life years (QALYs), a metric used broadly in the health economics literature (Neumann et al., 2015). We estimated QALYs for PCD patients as (Prieto and Sacristán, 2003):

$$QALYs={LE}_{PCD}*{HU}_{PCD}$$

Where LE_PCD_ is the Life Expectancy for PCD patients and HU_PCD_ is the Health Utility for PCD. The total QALYs saved for each algorithm were calculated using the relationship:

$$Total QALYs=TP*{PCD}_{treated}QALYs+FN*{PCD}_{not treated}QALYs$$

Where TP and FN are the number of tested patients characterized respectively as True Positive and False Negative per year, while PCD_treated_ QALYs are the number of QALYs saved when a PCD patient receives PCD-specific care and PCD_not treated_ QALYs are the number of QALYs saved when a PCD patients does not receive PCD-specific care. The treatment procedures followed in PCD management were derived from recent reviews and consensus papers from Europe and North America and calculation of annual treatment cost for an average diagnosed PCD patient was possible. (Barbato et al., 2009; Lucas et al., 2014; Shapiro et al., 2016; Strippoli et al., 2012) However there are no data regarding the treatment received by undiagnosed PCD patients and calculation of treatment cost for this subset of patients is not feasible. To overcome this lack of evidence we also performed a sensitivity analysis that provided a range of model results (CER and ICERs) while allowing the treatment cost for missed PCD patients to range from 3 times lower to 3 times higher as compared to the treatment cost for correctly diagnosed PCD patients. This sensitivity analysis reflects both the possible scenario of a PCD patient who has not been diagnosed and does not adhere to PCD specific treatment protocols, causing reduced healthcare costs as a result of, among other, less clinical appointments and physiotherapy consultations, reduced prescription of prophylactic antibiotics and less CT scans and the possible scenario of the same non-diagnosed patient causing higher healthcare costs due to higher frequency of exacerbations that may result to a higher frequency of hospitalizations and intravenous antibiotic administration, unnecessary tests and surgical procedures (e.g. grommets insertion, lung resection or lung transplantation) that could have otherwise been avoided (Yiallouros et al., 2015).

**Model parameters used in the secondary analysis**

In the extended model, estimates about the QALYs saved in PCD and the treatment cost as well as loss of productivity involved in PCD were required. Estimates about QALYs saved due to a specific treatment are usually available following randomized clinical trials (RCT) and life-long follow up of patients to inform about the life expectancy of the disease. In the case of PCD, RCT data are still scarce and information about life expectancy in PCD is non- existent. However, since life expectancy in many PCD patients is assumed to be normal or near-normal (Ferkol and Leigh, 2006; Kuehni et al., 2016) and using health utility (HU) estimates from other diseases with similar symptoms with PCD such as Cystic Fibrosis (CF) (HU ~ 0.75) (Bradley et al., 2013), chronic bronchitis and chronic sinusitis (HU ~ 0.80) (Fryback et al., 1993) and chronic obstructive pulmonary disease (HU ~ 0.85) (Stavem, 1999), a calculation regarding the number of QALYs saved in a PCD patient was possible. The calculation relies on the assumption that out of a full life expectancy of 80 years, 5 years (95%CI: 1.5-8.5) are lost due to PCD and that HU_PCD_ equals approximately 0.85 (95% CI: 0.80-0.0.90).

The cost of the different resources (i.e. physiotherapy equipment and consultations, specialist consultations, antibiotic administration, hearing aids) that are part of PCD specific treatment were obtained through national and international databases (NHS Reference Costs (National Health Service, ), Germany Federal Health Monitoring (Federal Health Monitoring, ), World Health Organization CHOICE database (World Health Organization, )). The cost of loss of productivity was calculated based on estimates of the cost of sick leaves from work available from Cystic Fibrosis literature (Chevreul K et al., 2016) allowing for milder disease burden (50% reduction) for adult PCD compared to adult CF patients. We did not include disability benefits or caregiver costs for adult PCD patients as these are very rare according to the authors’ experience. For all resources, we aimed to calculate European average resource prices by averaging prices from 3 countries (UK, Germany and Italy or Greece) and resource use was based on the experience of the authors. Nevertheless, a series of one-way sensitivity analyses, where individual parameters varied over their range, were carried out to test the robustness of our findings. A summary of the estimated treatment cost parameters that were included in the extended model are presented in Supplementary Table 1.

**Secondary Analysis Results**

The development of the extended model, allowed the calculation of ICERs which included the effectiveness and cost of PCD specific treatment as well. Supplementary Table 2 presents the lifetime effect (in QALYs saved) and resulting CER for the cohort of 1000 referrals. With the extended model, the most effective algorithm was nNO/HSVM+TEM with a mean of 6273 QALYs saved for the whole cohort followed by nNO+HSVM with 5521 QALYs and nNO+TEM with 4077 QALYs. The most cost-effective algorithm was nNO/HSVM+TEM followed by nNO+HSVM and nNO+TEM with mean CERs equal to €6,674/QALY saved, €8,944/QALY saved and €12,930/QALY saved respectively. The sensitivity analysis that allowed the ratio of treatment cost between undiagnosed PCD patient and diagnosed PCD patient to vary between 0.3 and 3 demonstrated that nNO/HSVM+TEM was always the most cost-effective algorithm but the differences in resulting CERs were greater when treatment cost for an undiagnosed PCD patient far exceeded the treatment cost for a diagnosed PCD patient (Supplementary Figure 1, absolute numbers available in Supplementary Table 3). The parameters that had the most important impact on the CER value of nNO/HSVM+TEM in the one-way sensitivity analyses, were PCD Health Utility and antibiotics cost, followed by reduction in life expectancy, loss of productivity and specialist visit cost. HSVM and TEM sensitivity had a much lower impact on the result as well as all other parameters (Supplementary Figure 2).

**Supplementary Table 1**: Estimated treatment costs for an average PCD patient in European Union

| **Treatment Costs** | **Mean**  **Resource Cost** | **Mean**  **Resource Use** |
| --- | --- | --- |
| Specialist Visit | €200/visit | 3 visits/year |
| Outpatient Visit | €65/visit | 3 visits/year |
| Antibiotics administration prophylaxis | Azithromycin (€22) | 6 times (prophylaxis)/year |
| Antibiotics administration exacerbation | Tobramycin (€2000) | Once/year |
| Other drug administration | Bronchodilators (€40) | 6 times/year |
| Physiotherapy consultation | €305 per consultation | Once/year |
| Sinus Surgery | €1880 per sinus surgery | 1 time/lifetime |
| Lung Transplantation | €38350 per transplantation | Very rare |
| Hospitalization Cost | €600 per bed-day | 2 bed days/year |
| Sputum Culture | €22 per sputum culture | 3 times/year |
| High Resolution Computed Tomography | €100 per HRCT | 3 times/lifetime |
| Pulmonary Function Testing | €190 per test | 3 times/year |
| Audiometry | €85 per test | 5 times/lifetime |
| Lung surgery | €20000 per surgery | Rare |
| Physiotherapy equipment | €200 per piece | Rare |
| Hearing aids | €500 per piece | Rare |
| Emergency room visit | €500 per visit | Rare |
| Home Oxygen Therapy | €200 per month | Rare |
| Sick leave | €1070 per year [1] | Common |

Sources: NHS Reference Costs (National Health Service, UK), Federal Health Monitoring (Germany), World Health Organization CHOICE database (World Health Organization)

**Supplementary Table 2: Total lifetime costs, total QALYs saved for a cohort of 1000 referrals (baseline analysis)**

| **Diagnostic Algorithm** | **QALYs saved**  **(95% CI)** | **CER (€/QALY saved)**  **(95% CI)*** |
| --- | --- | --- |
| **QALY** | 0 | - |
| **NO+TEM** | 4,077 (1732-5463) | 12,930 (7416-19440) |
| **NO+HSVM** | 5,521 (2291-7429) | 8,944 (5860-12970) |
| **NO/HSVM+TEM** | 6,273 (4394-8206) | 6,674 (5262-9454) |

*Compared to not performing any diagnostic procedure, CER: Average Cost-effectiveness Ratio

**Supplementary Table 3: Total lifetime costs, total QALYs saved for a cohort of 1000 referrals (sensitivity analysis)**

| Diagnostic Algorithm | Total Lifetime Cost in million € (95% CI) | | QALYs saved  (95% CI) | ACER (€/QALY saved)  (95% CI)* | |
| --- | --- | --- | --- | --- | --- |
| Ratio of undiagnosed PCD TC to diagnosed PCD TC | 0.3 | 3 | - | 0.3 | 3 |
| Do nothing | 0 | 0 | 0 | - | - |
| NO+TEM | 29.73  (18.48-34.80) | 71.63  (57.48-89.73) | 4077  (1732-5463) | 7,614  (5,932-11010) | 20,410  (13,420-32,630) |
| NO+HSVM | 36.45  (21.08-42.82) | 52.37  (40.73-76.88) | 5521  (2291-7429) | 6,889  (5,342-9694) | 11,890  (6,971-20,950) |
| NO/HSVM+TEM | 40.05  (31.98-47.31) | 42.38  (33.98-61.16) | 6273  (4394-8206) | 6,480  (5,193-8242) | 6,942  (5,266-12,350) |

* Compared to not performing any diagnostic procedure, CER: Average Cost-effectiveness Ratio, TC: lifetime treatment cost

**Supplementary Figure 1:** Sensitivity analysis for treatment cost in undiagnosed PCD


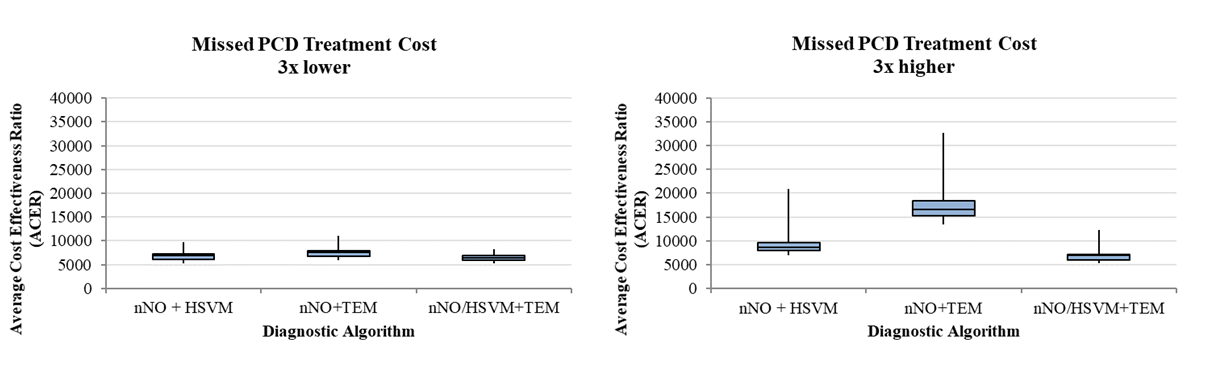


**Supplementary Figure 2: One-way sensitivity analyses for NO/HSVM+TEM CER (secondary analysis).** Tornado diagram demonstrating one-way sensitivity analyses of modelled parameters that affect the CER. The dashed vertical black line represents the base case value (CER=6,674 Euros/QALY saved). PCD: Primary Ciliary Dyskinesia, nNO: nasal Nitric Oxide, HSVM=High Speed Video Microscopy, ICER=incremental cost-effectiveness ratio.


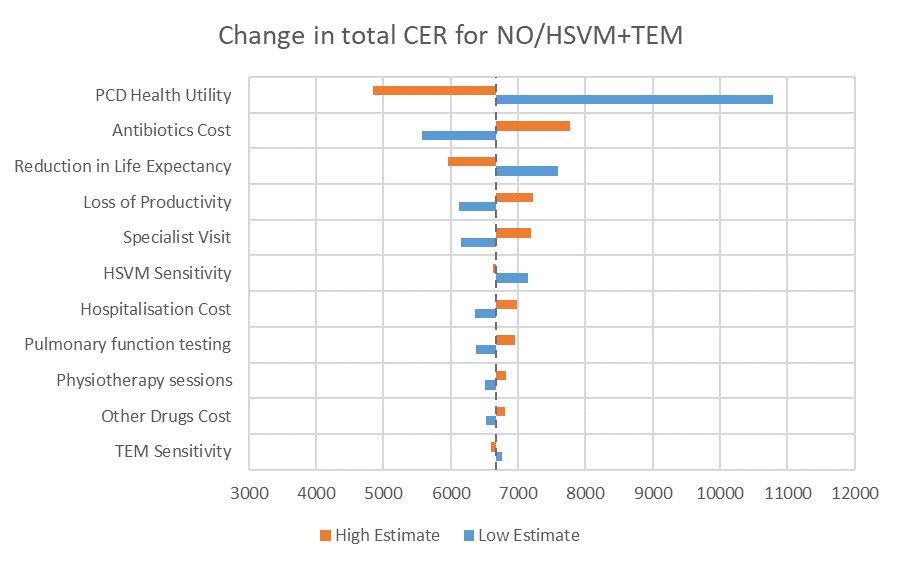


References:

1. Chevreul K, Michel M, Brigham, KB, López-Bastida J, Linertová R, Oliva-Moreno J, Serrano-Aguilar P, Posada-de-la-Paz M, Taruscio D, Schieppati A, Iskrov G, Pentek M, von der Schulenburg JMG Kanavos P, Persson U, Fattore G, BURQOL-RD Research Network. **Social/economic costs and health-related quality of life in patients with cystic fibrosis in Europe.** 2016 *The European Journal of Health Economics*, 17(1), 7-18.
